# 1,4-Di-*n*-hept­yloxy-2,5-dinitro­benzene

**DOI:** 10.1107/S160053680905123X

**Published:** 2009-12-04

**Authors:** Octavia A. Blackburn, Benjamin J. Coe, Robert Futhey, Madeleine Helliwell

**Affiliations:** a School of Chemistry, University of Manchester, Manchester M13 9PL, England

## Abstract

The complete molecule of the title compound, C_20_H_32_N_2_O_6_,  is generated by crystallographic inversion symmetry. The two mutually *trans* nitro substituents are hence in fully eclipsed conformation and also twisted by 43.2 (2)° with respect to the phenyl ring plane. The benzene-connected portions of the alk­oxy substituents lie almost coplanar with the ring [C—O—C—C torsion angle = 2.0 (2)°]. In the crystal, weak C—H⋯O interactions link the molecules.

## Related literature

For general background to the synthesis, see: Baker *et al.* (2008 ▶); Fisher *et al.* (1975 ▶); Flader *et al.* (2000 ▶); Hammershøj *et al.* (2006 ▶); Kawai *et al.* (1959 ▶). For a related structure, see: Voss *et al.* (2003 ▶).
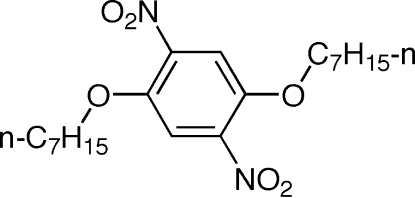


## Experimental

### 

#### Crystal data

C_20_H_32_N_2_O_6_
*M* *_r_* = 396.48
Monoclinic, 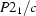

*a* = 13.988 (2) Å
*b* = 7.9454 (13) Å
*c* = 9.5344 (15) Å
β = 99.786 (3)°
*V* = 1044.3 (3) Å^3^
*Z* = 2
Mo *K*α radiation
μ = 0.09 mm^−1^
*T* = 100 K
0.40 × 0.40 × 0.25 mm

#### Data collection

Bruker SMART CCD area-detector diffractometer
5776 measured reflections
2110 independent reflections
1733 reflections with *I* > 2σ(*I*)
*R*_int_ = 0.035

#### Refinement

*R*[*F* ^2^ > 2σ(*F* ^2^)] = 0.035
*wR*(*F* ^2^) = 0.093
*S* = 1.03
2110 reflections
128 parameters
H-atom parameters constrained
Δρ_max_ = 0.24 e Å^−3^
Δρ_min_ = −0.19 e Å^−3^

### 

Data collection: *SMART* (Bruker, 2001 ▶); cell refinement: *SAINT* (Bruker, 2002 ▶); data reduction: *SAINT*; program(s) used to solve structure: *SHELXS97* (Sheldrick, 2008 ▶); program(s) used to refine structure: *SHELXL97* (Sheldrick, 2008 ▶); molecular graphics: *SHELXTL* (Sheldrick, 2008 ▶); software used to prepare material for publication: *SHELXTL* .

## Supplementary Material

Crystal structure: contains datablocks I, global. DOI: 10.1107/S160053680905123X/pv2242sup1.cif

Structure factors: contains datablocks I. DOI: 10.1107/S160053680905123X/pv2242Isup2.hkl

Additional supplementary materials:  crystallographic information; 3D view; checkCIF report

## Figures and Tables

**Table 1:** Hydrogen-bond geometry (Å, °)

*D*—H⋯*A*	*D*—H	H⋯*A*	*D*⋯*A*	*D*—H⋯*A*
C3—H3⋯O2^i^	0.95	2.50	3.4525 (15)	179
C4—H4*B*⋯O1^ii^	0.99	2.53	3.2852 (15)	133

Symmetry codes: (i) 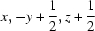; (ii) 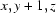.

**Fig. 1.**
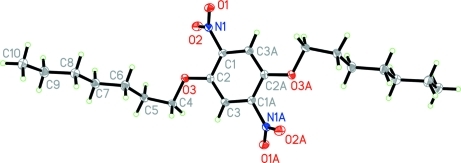
View of the compound (I) (50% probability displacement ellipsoids); [symmetry code: A = -x + 1, -y, -z + 2].

**Fig. 2.**
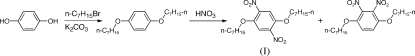
Synthesis of compound (I) and its isomeric form.

## supplementary crystallographic information

### Comment

The title compound, (I), is the minor product formed from the nitration of 1,4-di(n-heptoxy)benzene and was synthesized as a precursor to derivatized "salen-like" ligands for co-ordination to transition metals. Although (I) is commercially available, apparently a synthetic method has not been reported previously. Our synthesis involves a standard nitration procedure (Hammershøj *et al.*, 2006) and produces a mixture of the 2,3 and 2,5 structural isomers in a *ca* 2:1 ratio as indicated by the ^1^H NMR spectrum of the crude material. The isomeric ratio produced in such reactions is clearly quite variable. For example, nitration of 1,4-dimethoxybenzene by a very similar method, but with heating at 373 K for 1 h produced the 2,3 isomer in 90% yield after recrystallization (Hammershøj *et al.*, 2006). Similar results were reported previously (Flader *et al.*, 2000; Fisher *et al.*, 1975), while nitration of 1,4-di(n-butoxy)benzene in a mixture of nitric and acetic acids gives the 2,3 and 2,5 isomers in a 4:1 ratio (Kawai *et al.*, 1959; Baker *et al.*, 2008).

Having structural confirmation for (I), the two isomers are also distinguished by significant differences in their ^1^H NMR spectra, especially a high field shift of 0.36 p.p.m. for the singlet assigned to the two phenyl protons on moving from 2,5 to 2,3-isomer. This change can be attributed to an increased extent of shielding when these protons are located *meta* rather than *ortho* to the nitro substituents. The two isomers also show significantly different melting points and electronic absorption spectra. Compound (I) melts at a temperature *ca* 70 K higher than that observed for its 2,3-isomer, indicating that the forces holding together the crystal lattice are considerably stronger for (I). A similarly large difference in melting points has also been reported for the corresponding n-butoxy compounds (Kawai *et al.*, 1959).

Both isomers show relatively intense near UV absorption bands that are responsible for their observed colours. These bands are attributable to π→π* intramolecular charge-transfer (ICT) excitations from the HOMO primarily localized on the electron-rich heptoxy groups to the LUMO localized on the electron-deficient nitro units. The stronger yellow colour of (I) when compared with its 2,3-isomer is due to the ICT band maximum being lower in energy by *ca* 940 cm^-1^, with an approximately doubled molar extinction coefficient, producing more extensive tailing of the absorption into the visible region. Clearly, both the HOMO-LUMO energy gap and the extent of overlap between these orbitals are affected significantly by isomerization.

Compound (I) readily forms large and high-quality, yellow block-shaped crystals upon slow evaporation of a n-hexane/ethyl acetate solution. Its structure (Fig. 1) resembles that reported previously for the compound 2-(n-heptoxy)-5-methoxy-3,6-dinitrobenzaldehyde (Voss *et al.*, 2003), with generally similar geometric parameters. In both compounds, the two mutually *trans* nitro substituents are twisted with respect to the phenyl ring plane. However, in (I) these groups are fully eclipsed, since they are related by inversion, each with a O2—N1—C1—C2 torsion angle of 43.2 (2)°, while their mutual orientation is staggered in the previously published structure, with corresponding angles of 39.3 (5) and 87.5 (4)°. Another difference between these two structures is the relative orientations of their alkoxy substituents. In (I), for the inversion-related alkoxy groups C4—O3—C2—C3, the torsion angles are very small (2.0 (2)°), but in 2-(n-heptoxy)-5-methoxy-3,6-dinitrobenzaldehyde, the C—O—C—C angles are quite different, being 1.0 (5)° for the methoxy substituent, while the OCH_2_ unit of the heptoxy group is almost perpendicular to the phenyl ring, with a C—O—C—C torsion angle of 86.9 (4)°.

### Experimental

Synthesis of 1,4-di(n-heptoxy)benzene. A solution of hydroquinone (5.00 g, 0.045 mol), 1-bromo-n-heptane (17.9 g, 0.100 mol) and K_2_CO_3_ (25.1 g, 0.182 mol) in DMF (100 ml) was heated at reflux for 3 h. The resulting brown solution was poured into cold water and the brown precipitate filtered off, washed with cold water and recrystallized from ethanol to give a colourless solid (yield 7.22 g, 52%).

Synthesis of 1,4-di(n-heptoxy)-2,5-dinitrobenzene (I). 1,4-di(n-heptoxy)benzene (200 mg, 0.653 mmol) was added slowly to stirred, ice cooled nitric acid (67%, 5 ml). The solution was stirred at 273 K for 1 h, at room temperature for 1 h and then at 313 K for 1 h. The reaction mixture was poured into iced water (10 g) and the product extracted into chloroform (10 ml). The yellow solution was dried over MgSO_4_ and evaporated to give a mixture of the 2,3-dinitro and 2,5-dinitro isomers as a yellow solid (yield 247 mg, 95%). The isomers were separated by silica gel column chromatography. Elution with n-hexane/ethyl acetate (99:1) gave (I) as a bright yellow solid (yield 71 mg, 27%). Diffraction-quality crystals were grown by slow evaporation of a n-hexane/ethyl acetate solution. Further elution of the column with n-hexane/ethyl acetate (95:5) gave the 2,3-dinitro isomer as a pale yellow solid (yield 123 mg, 48%).

### Refinement

H atoms bonded to the C atoms were fixed geometrically and treated as riding with C—H = 0.95 Å (aromatic), 0.98 Å (methyl) and 0.99 Å (methylene), with *U*_iso_(H) = 1.2 times those of the parent atoms for the aromatic and methylene H atoms and *U*_iso_(H) = 1.5 times those of the parent atoms for the methyl H atoms.

### Crystal data

**Table:**

C_20_H_32_N_2_O_6_	*F*(000) = 428
*M**_r_* = 396.48	*D*_x_ = 1.261 Mg m^−^^3^
Monoclinic, *P*2_1_/*c*	Mo *K*α radiation, λ = 0.71073 Å
Hall symbol: -P 2ybc	Cell parameters from 776 reflections
*a* = 13.988 (2) Å	θ = 3.0–26.2°
*b* = 7.9454 (13) Å	µ = 0.09 mm^−^^1^
*c* = 9.5344 (15) Å	*T* = 100 K
β = 99.786 (3)°	Block, yellow
*V* = 1044.3 (3) Å^3^	0.40 × 0.40 × 0.25 mm
*Z* = 2	

### Data collection

**Table:**

Bruker SMART CCD area-detector diffractometer	1733 reflections with *I* > 2σ(*I*)
Radiation source: fine-focus sealed tube	*R*_int_ = 0.035
graphite	θ_max_ = 26.3°, θ_min_ = 3.0°
phi and ω scans	*h* = −15→17
5776 measured reflections	*k* = −9→9
2110 independent reflections	*l* = −8→11

### Refinement

**Table:**

Refinement on *F*^2^	Primary atom site location: structure-invariant direct methods
Least-squares matrix: full	Secondary atom site location: difference Fourier map
*R*[*F*^2^ > 2σ(*F*^2^)] = 0.035	Hydrogen site location: inferred from neighbouring sites
*wR*(*F*^2^) = 0.093	H-atom parameters constrained
*S* = 1.03	*w* = 1/[σ^2^(*F*_o_^2^) + (0.0502*P*)^2^ + 0.0454*P*] where *P* = (*F*_o_^2^ + 2*F*_c_^2^)/3
2110 reflections	(Δ/σ)_max_ < 0.001
128 parameters	Δρ_max_ = 0.24 e Å^−^^3^
0 restraints	Δρ_min_ = −0.19 e Å^−^^3^

### Special details

**Table:**

Experimental. Characterization data for 1,4-di(n-heptoxy)benzene. Found: C 78.76, H 11.14%. Calculated for C_20_H_34_O_2_: C 78.38, H, 11.18%. ^1^H NMR (300 MHz, CDCl_3_): 6.82 (4H, s, C_6_H_4_), 3.89 (4H, t, J = 6.6 Hz, 2OCH_2_), 1.75 (4H, quintet, J = 6.7 Hz, 2CH_2_), 1.50-1.18 (16H, m, 8CH_2_), 0.89 (6H, t, J = 6.8 Hz, 2CH_3_).Characterization data for 1,4-di(n-heptoxy)-2,5-dinitrobenzene (I). Melting point = 387-389 K. Found: C 60.81, H 8.29, N 6.94%. Calculated for C_20_H_32_N_2_O_6_: C 60.59, H 8.14, N 7.07%. ^1^H NMR (400 MHz, CDCl_3_): 7.51 (2H, s, C_6_H_2_), 4.08 (4H, t, J = 6.5 Hz, 2OCH_2_), 1.83 (4H, quintet, J = 6.6 Hz, 2CH_2_), 1.50-1.25 (16H, m, 8CH_2_), 0.89 (6H, t, J = 6.7 Hz, 2CH_3_). +Electrospray MS: m/z = 419.2 [M + Na]^+^, 815.8 [2M + Na]^+^. (λ_max_ = 378 nm, ε = 5000 M^-1^ dm^3^ in dichloromethane). ν(NO_2_) = 1531 and 1352 cm^-1^.Characterization data for 1,4-di(n-heptoxy)-2,3-dinitrobenzene. Melting point = 318-319 K. Found: C 60.66, H 8.56, N 7.09%. Calculated for C_20_H_32_N_2_O_6_: C 60.59, H 8.14, N 7.07%. ^1^H NMR (400 MHz, CDCl_3_): 7.15 (2H, s, C_6_H_2_), 4.05 (4H, t, J = 6.5 Hz, 2OCH_2_), 1.76 (4H, quintet, J = 6.5 Hz, 2CH_2_), 1.45-1.25 (16H, m, 8CH_2_), 0.89 (6H, t, J = 6.7 Hz, 2CH_3_). (λ_max_ = 365 nm, ε = 2300 M^-1^ dm^3^ in dichloromethane). ν(NO_2_) = 1537 and 1358 cm^-1^.
Geometry. All e.s.d.'s (except the e.s.d. in the dihedral angle between two l.s. planes) are estimated using the full covariance matrix. The cell e.s.d.'s are taken into account individually in the estimation of e.s.d.'s in distances, angles and torsion angles; correlations between e.s.d.'s in cell parameters are only used when they are defined by crystal symmetry. An approximate (isotropic) treatment of cell e.s.d.'s is used for estimating e.s.d.'s involving l.s. planes.
Refinement. Refinement of *F*^2^ against ALL reflections. The weighted *R*-factor *wR* and goodness of fit *S* are based on *F*^2^, conventional *R*-factors *R* are based on *F*, with *F* set to zero for negative *F*^2^. The threshold expression of *F*^2^ > σ(*F*^2^) is used only for calculating *R*-factors(gt) *etc*. and is not relevant to the choice of reflections for refinement. *R*-factors based on *F*^2^ are statistically about twice as large as those based on *F*, and *R*- factors based on ALL data will be even larger.

### Fractional atomic coordinates and isotropic or equivalent isotropic displacement parameters (Å^2^)

**Table:**

	*x*	*y*	*z*	*U*_iso_*/*U*_eq_	
O1	0.60328 (6)	−0.29610 (10)	0.79050 (10)	0.0209 (2)	
O2	0.58901 (6)	−0.06141 (10)	0.67325 (9)	0.0233 (2)	
O3	0.63728 (6)	0.17278 (10)	0.87825 (9)	0.0194 (2)	
N1	0.58118 (7)	−0.14678 (12)	0.77792 (11)	0.0158 (2)	
C1	0.54045 (8)	−0.06789 (14)	0.89396 (12)	0.0146 (3)	
C2	0.56913 (8)	0.09431 (15)	0.94007 (12)	0.0153 (3)	
C3	0.52640 (8)	0.16163 (14)	1.04846 (13)	0.0156 (3)	
H3	0.5431	0.2718	1.0830	0.019*	
C4	0.66991 (9)	0.33643 (14)	0.93230 (13)	0.0179 (3)	
H4A	0.6913	0.3318	1.0367	0.022*	
H4B	0.6168	0.4198	0.9110	0.022*	
C5	0.75354 (8)	0.38479 (15)	0.85918 (13)	0.0187 (3)	
H5A	0.7716	0.5033	0.8825	0.022*	
H5B	0.7324	0.3770	0.7549	0.022*	
C6	0.84229 (9)	0.27363 (16)	0.90244 (15)	0.0226 (3)	
H6A	0.8267	0.1586	0.8657	0.027*	
H6B	0.8565	0.2669	1.0076	0.027*	
C7	0.93312 (9)	0.33431 (16)	0.84913 (14)	0.0217 (3)	
H7A	0.9219	0.3296	0.7439	0.026*	
H7B	0.9457	0.4531	0.8779	0.026*	
C8	1.02172 (9)	0.22911 (16)	0.90717 (15)	0.0236 (3)	
H8A	1.0301	0.2290	1.0124	0.028*	
H8B	1.0095	0.1116	0.8746	0.028*	
C9	1.11579 (9)	0.28829 (18)	0.86375 (15)	0.0263 (3)	
H9A	1.1257	0.4087	0.8887	0.032*	
H9B	1.1104	0.2779	0.7592	0.032*	
C10	1.20347 (10)	0.18840 (18)	0.93540 (16)	0.0292 (3)	
H10A	1.2101	0.2003	1.0389	0.044*	
H10B	1.2620	0.2312	0.9039	0.044*	
H10C	1.1947	0.0694	0.9095	0.044*	

### Atomic displacement parameters (Å^2^)

**Table:**

	*U*^11^	*U*^22^	*U*^33^	*U*^12^	*U*^13^	*U*^23^
O1	0.0233 (5)	0.0140 (5)	0.0262 (5)	0.0037 (3)	0.0066 (4)	0.0002 (4)
O2	0.0337 (5)	0.0197 (5)	0.0184 (5)	−0.0009 (4)	0.0104 (4)	0.0022 (4)
O3	0.0213 (5)	0.0162 (4)	0.0226 (5)	−0.0066 (3)	0.0095 (4)	−0.0030 (4)
N1	0.0154 (5)	0.0146 (5)	0.0175 (5)	−0.0010 (4)	0.0030 (4)	−0.0002 (4)
C1	0.0152 (6)	0.0152 (6)	0.0133 (6)	0.0025 (5)	0.0021 (5)	0.0008 (5)
C2	0.0139 (6)	0.0155 (6)	0.0161 (6)	−0.0002 (5)	0.0016 (5)	0.0030 (5)
C3	0.0165 (6)	0.0125 (6)	0.0167 (6)	−0.0002 (4)	−0.0002 (5)	0.0005 (5)
C4	0.0201 (6)	0.0136 (6)	0.0200 (6)	−0.0031 (5)	0.0032 (5)	−0.0013 (5)
C5	0.0196 (6)	0.0166 (6)	0.0200 (6)	−0.0042 (5)	0.0038 (5)	0.0010 (5)
C6	0.0214 (7)	0.0212 (7)	0.0263 (7)	−0.0012 (5)	0.0069 (6)	0.0033 (5)
C7	0.0209 (7)	0.0252 (7)	0.0191 (7)	−0.0031 (5)	0.0041 (5)	0.0003 (5)
C8	0.0219 (7)	0.0253 (7)	0.0244 (7)	−0.0018 (5)	0.0060 (6)	−0.0024 (6)
C9	0.0214 (7)	0.0366 (8)	0.0215 (7)	−0.0039 (6)	0.0050 (6)	−0.0019 (6)
C10	0.0220 (7)	0.0349 (8)	0.0319 (8)	−0.0012 (6)	0.0079 (6)	−0.0063 (6)

### Geometric parameters (Å, °)

**Table:**

O1—N1	1.2268 (12)	C6—C7	1.5247 (16)
O2—N1	1.2269 (12)	C6—H6A	0.9900
O3—C2	1.3549 (13)	C6—H6B	0.9900
O3—C4	1.4438 (14)	C7—C8	1.5191 (18)
N1—C1	1.4682 (14)	C7—H7A	0.9900
C1—C3^i^	1.3803 (16)	C7—H7B	0.9900
C1—C2	1.3983 (16)	C8—C9	1.5196 (17)
C2—C3	1.3862 (16)	C8—H8A	0.9900
C3—C1^i^	1.3804 (16)	C8—H8B	0.9900
C3—H3	0.9500	C9—C10	1.5218 (19)
C4—C5	1.5097 (16)	C9—H9A	0.9900
C4—H4A	0.9900	C9—H9B	0.9900
C4—H4B	0.9900	C10—H10A	0.9800
C5—C6	1.5223 (17)	C10—H10B	0.9800
C5—H5A	0.9900	C10—H10C	0.9800
C5—H5B	0.9900		
			
C2—O3—C4	117.53 (9)	C5—C6—H6B	108.7
O2—N1—O1	123.94 (10)	C7—C6—H6B	108.7
O2—N1—C1	118.47 (9)	H6A—C6—H6B	107.6
O1—N1—C1	117.57 (9)	C8—C7—C6	112.28 (10)
C3^i^—C1—C2	123.36 (10)	C8—C7—H7A	109.1
C3^i^—C1—N1	116.43 (10)	C6—C7—H7A	109.1
C2—C1—N1	120.21 (10)	C8—C7—H7B	109.1
O3—C2—C3	124.83 (11)	C6—C7—H7B	109.1
O3—C2—C1	118.22 (10)	H7A—C7—H7B	107.9
C3—C2—C1	116.94 (10)	C9—C8—C7	114.90 (11)
C1^i^—C3—C2	119.70 (11)	C9—C8—H8A	108.5
C1^i^—C3—H3	120.1	C7—C8—H8A	108.5
C2—C3—H3	120.1	C9—C8—H8B	108.5
O3—C4—C5	106.69 (9)	C7—C8—H8B	108.5
O3—C4—H4A	110.4	H8A—C8—H8B	107.5
C5—C4—H4A	110.4	C8—C9—C10	112.67 (12)
O3—C4—H4B	110.4	C8—C9—H9A	109.1
C5—C4—H4B	110.4	C10—C9—H9A	109.1
H4A—C4—H4B	108.6	C8—C9—H9B	109.1
C4—C5—C6	112.80 (10)	C10—C9—H9B	109.1
C4—C5—H5A	109.0	H9A—C9—H9B	107.8
C6—C5—H5A	109.0	C9—C10—H10A	109.5
C4—C5—H5B	109.0	C9—C10—H10B	109.5
C6—C5—H5B	109.0	H10A—C10—H10B	109.5
H5A—C5—H5B	107.8	C9—C10—H10C	109.5
C5—C6—C7	114.44 (10)	H10A—C10—H10C	109.5
C5—C6—H6A	108.7	H10B—C10—H10C	109.5
C7—C6—H6A	108.7		
			
O2—N1—C1—C3^i^	136.39 (11)	N1—C1—C2—C3	178.96 (10)
O1—N1—C1—C3^i^	−41.95 (14)	O3—C2—C3—C1^i^	−178.21 (11)
O2—N1—C1—C2	−43.17 (15)	C1—C2—C3—C1^i^	0.53 (18)
O1—N1—C1—C2	138.49 (11)	C2—O3—C4—C5	172.90 (10)
C4—O3—C2—C3	2.00 (17)	O3—C4—C5—C6	−67.13 (13)
C4—O3—C2—C1	−176.73 (10)	C4—C5—C6—C7	−171.07 (11)
C3^i^—C1—C2—O3	178.27 (11)	C5—C6—C7—C8	174.35 (11)
N1—C1—C2—O3	−2.21 (16)	C6—C7—C8—C9	−177.18 (11)
C3^i^—C1—C2—C3	−0.56 (19)	C7—C8—C9—C10	174.81 (11)

Symmetry codes: (i) −*x*+1, −*y*, −*z*+2.

### Hydrogen-bond geometry (Å, °)

**Table:**

*D*—H···*A*	*D*—H	H···*A*	*D*···*A*	*D*—H···*A*
C3—H3···O2^ii^	0.95	2.50	3.4525 (15)	179
C4—H4B···O1^iii^	0.99	2.53	3.2852 (15)	133

Symmetry codes: (ii) *x*, −*y*+1/2, *z*+1/2; (iii) *x*, *y*+1, *z*.
